# Supplementary material for: HIVprotI: an integrated web based platform for prediction and design of HIV proteins inhibitors
Source: J Cheminform. 2018 Mar 9;10:12. doi: 10.1186/s13321-018-0266-y (PMC5845081; doi:10.1186/s13321-018-0266-y)
Supplement: Supplementary file 2 — Additional file 2. Source code of HIVProtI web server. [file 13321_2018_266_MOESM2_ESM.zip › HIVprotI_Source-code/hivprotI/weka-3-6-6/doc/weka/filters/unsupervised/attribute/Normalize.html]

Normalize


---


|  |  |  |
| --- | --- | --- |
| **Overview** \| **Package** \| **Class** \| **Tree** \| **Deprecated** \| **Index** \| **Help** \| **Weka's home** | |  |
| **PREV CLASS**   **NEXT CLASS** | **FRAMES**    **NO FRAMES**     **All Classes** |  |
| SUMMARY: NESTED \| FIELD \| CONSTR \| METHOD | DETAIL: FIELD \| CONSTR \| METHOD |  |


---


## weka.filters.unsupervised.attribute Class Normalize

```
java.lang.Object
  weka.filters.Filter
      weka.filters.unsupervised.attribute.PotentialClassIgnorer
          weka.filters.unsupervised.attribute.Normalize
```

**All Implemented Interfaces:**: java.io.Serializable, CapabilitiesHandler, OptionHandler, RevisionHandler, Sourcable, UnsupervisedFilter

---

``` public class Normalize extends PotentialClassIgnorer implements UnsupervisedFilter, Sourcable, OptionHandler ```

Normalizes all numeric values in the given dataset (apart from the class attribute, if set). The resulting values are by default in [0,1] for the data used to compute the normalization intervals. But with the scale and translation parameters one can change that, e.g., with scale = 2.0 and translation = -1.0 you get values in the range [-1,+1].


Valid options are:

```
 -unset-class-temporarily
  Unsets the class index temporarily before the filter is
  applied to the data.
  (default: no)
```

```
 -S <num>
  The scaling factor for the output range.
  (default: 1.0)
```

```
 -T <num>
  The translation of the output range.
  (default: 0.0)
```

**Version:**
:   $Revision: 5543 $

**Author:**
:   Eibe Frank (eibe@cs.waikato.ac.nz), FracPete (fracpete at waikato dot ac dot nz)

**See Also:**: Serialized Form

---

**Constructor Summary**

`Normalize()`


| **Method Summary** | |
| --- | --- |
| `boolean` | `batchFinished()`             Signify that this batch of input to the filter is finished. |
| `Capabilities` | `getCapabilities()`             Returns the Capabilities of this filter. |
| `double[]` | `getMaxArray()`             Returns the calculated maximum values for the attributes in the data. |
| `double[]` | `getMinArray()`             Returns the calculated minimum values for the attributes in the data. |
| `java.lang.String[]` | `getOptions()`             Gets the current settings of the filter. |
| `java.lang.String` | `getRevision()`             Returns the revision string. |
| `double` | `getScale()`             Get the scaling factor. |
| `double` | `getTranslation()`             Get the translation. |
| `java.lang.String` | `globalInfo()`             Returns a string describing this filter. |
| `boolean` | `input(Instance instance)`             Input an instance for filtering. |
| `java.util.Enumeration` | `listOptions()`             Returns an enumeration describing the available options. |
| `static void` | `main(java.lang.String[] args)`             Main method for running this filter. |
| `java.lang.String` | `scaleTipText()`             Returns the tip text for this property. |
| `boolean` | `setInputFormat(Instances instanceInfo)`             Sets the format of the input instances. |
| `void` | `setOptions(java.lang.String[] options)`             Parses a given list of options. |
| `void` | `setScale(double value)`             Sets the scaling factor. |
| `void` | `setTranslation(double value)`             Sets the translation. |
| `java.lang.String` | `toSource(java.lang.String className, Instances data)`             Returns a string that describes the filter as source. |
| `java.lang.String` | `translationTipText()`             Returns the tip text for this property. |

**Methods inherited from class weka.filters.unsupervised.attribute.PotentialClassIgnorer**

`getIgnoreClass, getOutputFormat, ignoreClassTipText, setIgnoreClass`

**Methods inherited from class weka.filters.Filter**

`batchFilterFile, filterFile, getCapabilities, isFirstBatchDone, isNewBatch, isOutputFormatDefined, makeCopies, makeCopy, numPendingOutput, output, outputPeek, toString, useFilter, wekaStaticWrapper`

**Methods inherited from class java.lang.Object**

`equals, getClass, hashCode, notify, notifyAll, wait, wait, wait`

**Constructor Detail**

### Normalize

```
public Normalize()
```


**Method Detail**

### globalInfo

```
public java.lang.String globalInfo()
```

:   Returns a string describing this filter.

    :   **Returns:**: a description of the filter suitable for displaying in the explorer/experimenter gui

---


### listOptions

```
public java.util.Enumeration listOptions()
```

:   Returns an enumeration describing the available options.

    :   **Specified by:**: `listOptions` in interface `OptionHandler` **Overrides:**: `listOptions` in class `PotentialClassIgnorer`
    :   **Returns:**: an enumeration of all the available options.

---


### setOptions

```
public void setOptions(java.lang.String[] options)
                throws java.lang.Exception
```

:   Parses a given list of options.


    Valid options are:

    ```
     -unset-class-temporarily
      Unsets the class index temporarily before the filter is
      applied to the data.
      (default: no)
    ```

    ```
     -S <num>
      The scaling factor for the output range.
      (default: 1.0)
    ```

    ```
     -T <num>
      The translation of the output range.
      (default: 0.0)
    ```

    :   **Specified by:**: `setOptions` in interface `OptionHandler` **Overrides:**: `setOptions` in class `PotentialClassIgnorer`
    :   **Parameters:**: `options` - the list of options as an array of strings **Throws:**: `java.lang.Exception` - if an option is not supported

---


### getOptions

```
public java.lang.String[] getOptions()
```

:   Gets the current settings of the filter.

    :   **Specified by:**: `getOptions` in interface `OptionHandler` **Overrides:**: `getOptions` in class `PotentialClassIgnorer`
    :   **Returns:**: an array of strings suitable for passing to setOptions

---


### getCapabilities

```
public Capabilities getCapabilities()
```

:   Returns the Capabilities of this filter.

    :   **Specified by:**: `getCapabilities` in interface `CapabilitiesHandler` **Overrides:**: `getCapabilities` in class `Filter`
    :   **Returns:**: the capabilities of this object **See Also:**: `Capabilities`

---


### setInputFormat

```
public boolean setInputFormat(Instances instanceInfo)
                       throws java.lang.Exception
```

:   Sets the format of the input instances.

    :   **Overrides:**: `setInputFormat` in class `PotentialClassIgnorer`
    :   **Parameters:**: `instanceInfo` - an Instances object containing the input instance structure (any instances contained in the object are ignored - only the structure is required). **Returns:**: true if the outputFormat may be collected immediately **Throws:**: `java.lang.Exception` - if the input format can't be set successfully

---


### input

```
public boolean input(Instance instance)
              throws java.lang.Exception
```

:   Input an instance for filtering. Filter requires all
    training instances be read before producing output.

    :   **Overrides:**: `input` in class `Filter`
    :   **Parameters:**: `instance` - the input instance **Returns:**: true if the filtered instance may now be collected with output(). **Throws:**: `java.lang.Exception` - if an error occurs: `java.lang.IllegalStateException` - if no input format has been set.

---


### batchFinished

```
public boolean batchFinished()
                      throws java.lang.Exception
```

:   Signify that this batch of input to the filter is finished.
    If the filter requires all instances prior to filtering,
    output() may now be called to retrieve the filtered instances.

    :   **Overrides:**: `batchFinished` in class `Filter`
    :   **Returns:**: true if there are instances pending output **Throws:**: `java.lang.Exception` - if an error occurs: `java.lang.IllegalStateException` - if no input structure has been defined

---


### toSource

```
public java.lang.String toSource(java.lang.String className,
                                 Instances data)
                          throws java.lang.Exception
```

:   Returns a string that describes the filter as source. The
    filter will be contained in a class with the given name (there may
    be auxiliary classes),
    and will contain two methods with these signatures:

    ```
     // converts one row
     public static Object[] filter(Object[] i);
     // converts a full dataset (first dimension is row index)
     public static Object[][] filter(Object[][] i);
    ```

    where the array `i` contains elements that are either
    Double, String, with missing values represented as null. The generated
    code is public domain and comes with no warranty.

    :   **Specified by:**: `toSource` in interface `Sourcable`
    :   **Parameters:**: `className` - the name that should be given to the source class.: `data` - the dataset used for initializing the filter **Returns:**: the object source described by a string **Throws:**: `java.lang.Exception` - if the source can't be computed

---


### getMinArray

```
public double[] getMinArray()
```

:   Returns the calculated minimum values for the attributes in the data.

    :   **Returns:**: the array with the minimum values

---


### getMaxArray

```
public double[] getMaxArray()
```

:   Returns the calculated maximum values for the attributes in the data.

    :   **Returns:**: the array with the maximum values

---


### scaleTipText

```
public java.lang.String scaleTipText()
```

:   Returns the tip text for this property.

    :   **Returns:**: tip text for this property suitable for displaying in the explorer/experimenter gui

---


### getScale

```
public double getScale()
```

:   Get the scaling factor.

    :   **Returns:**: the factor

---


### setScale

```
public void setScale(double value)
```

:   Sets the scaling factor.

    :   **Parameters:**: `value` - the scaling factor

---


### translationTipText

```
public java.lang.String translationTipText()
```

:   Returns the tip text for this property.

    :   **Returns:**: tip text for this property suitable for displaying in the explorer/experimenter gui

---


### getTranslation

```
public double getTranslation()
```

:   Get the translation.

    :   **Returns:**: the translation

---


### setTranslation

```
public void setTranslation(double value)
```

:   Sets the translation.

    :   **Parameters:**: `value` - the translation

---


### getRevision

```
public java.lang.String getRevision()
```

:   Returns the revision string.

    :   **Specified by:**: `getRevision` in interface `RevisionHandler` **Overrides:**: `getRevision` in class `Filter`
    :   **Returns:**: the revision

---


### main

```
public static void main(java.lang.String[] args)
```

:   Main method for running this filter.

    :   **Parameters:**: `args` - should contain arguments to the filter, use -h for help


---


|  |  |  |
| --- | --- | --- |
| **Overview** \| **Package** \| **Class** \| **Tree** \| **Deprecated** \| **Index** \| **Help** \| **Weka's home** | |  |
| **PREV CLASS**   **NEXT CLASS** | **FRAMES**    **NO FRAMES**     **All Classes** |  |
| SUMMARY: NESTED \| FIELD \| CONSTR \| METHOD | DETAIL: FIELD \| CONSTR \| METHOD |  |


---
